# Introduction to Peptidyl-Prolyl *cis/trans* Isomerase (PPIase) Series †

**DOI:** 10.3390/biom9020074

**Published:** 2019-02-20

**Authors:** Andrzej Galat

**Affiliations:** Retired from: Service d’Ingénierie Moléculaire des Protéines (SIMOPRO), CEA-Université Paris–Saclay, 91191 Gif-sur-Yvette, France; andrzej.galat@sfr.fr

About 30 years after the discovery of peptidyl-prolyl *cis*/*trans* isomerases (PPIases), research on this group of proteins has become somewhat calmer than it used to be, but it still generates lots of interest. Thereby we decided to present a series of reviews on some novel aspects of PPIases expressed in various organisms. Several research groups from around the world agreed to present some recent developments in the field of PPIases.

Research on FK506-binding proteins (FKBPs) began at the end of the 1980s in the laboratory of Professor Stuart L. Schreiber, at the Department of Chemistry, Harvard University (Cambridge, MA, USA). I started a pioneering work on the purification and characterization of a potential factor present in different organs, which could bind to the novel (at that time) immunosuppressive drug FK506 [1]. In a relatively short time, our collaborator, Dr. M. Harding, had isolated some amounts of a 12 kDa protein that was identical to the kind found on sodium dodecyl sulphate-polyacrylamide gel electrophoresis (SDS-PAGE) gels of proteins tightly bound to FK506-affinity gels made by Dr. D.E. Uehling in Professor Schreiber’s lab. This 12 kDa protein, soon named immunophilin, had PPIase activity, which was, to some extent, similar to another PPIase, namely cyclophilin that selectively binds the immunosuppressive drug cyclosporin A (CsA) [2]. By the end of 1989, the colleagues at the lab characterized the kinetics and substrate specificity of FKBP12 and its structure was predicted on the basis of circular dichroism data that was well confirmed by X-ray and nuclear magnetic resonance (NMR) spectroscopy. Since that time, a series of seminal discoveries made by other researchers in Professor Schreiber’s lab demonstrated that ternary and multiple-component complexes of FKBP12/FK506 or FKBP12/rapamycin were responsible for the immunosuppressive activity of these complexes in vivo [3,4,5].

The chapter given above could be accompanied by short personal accounts on how it still remains unforgettable, the fresh breeze of wind that I had followed on an old bike along the Charles River from Brookline to Cambridge every morning and back every evening. Because of a relatively long distance (15 km) each way, quite often I stopped at the grand French bar named “Au bon pain”, which was right on the Harvard square where they served big American coffee and all sorts of “délices et pâtisseries françaises des rêves”. The Harvard Square was a lively place where many restaurants and bars were opened late in to the night. There was a good German restaurant with splendid dishes, and diverse sandwich and coffee shops that served tasty and not so expensive food. Finally, I would be dishonest if I failed to mention a very tasty beer called Samuel Adams. All of the rich and quickly evolving research events kept me in a joyful spirit for more than two and a half years, despite any weather conditions in the winter (sometimes snowy and slippery), in the beautiful spring, and the hot Massachusetts summers. This is a brief story on how I became involved in the research on FKBPs. It is important to mention however that similar research was conducted by Dr. Siekerka and his colleagues at Merck Labs [6].

Almost since the beginning of our research, it became clear that PPIase activity of the FKBPs or cyclophilins were of lesser importance in comparison to their capacity to form complexes with some clinically-useful drugs, which could block enzymatic activity of minor components in the cell that causes immunosuppression. Later it was shown that some FKBPs as well as cyclophilins were parts of gigantic complexes, namely FKBP12b–ryanodine receptors or supramacromolecular entities such as diverse components of splicing machinery or gene transcriptional complexes in the cell. 

In this series of reviews, we present a splendid and well-illustrated analysis of nuclear cyclophilins, which participate in the regulation of various splicing events. This part was analyzed by Drs. C. Rajiv and T.L. Davis [7]. Research on mitochondrial small size cyclophilin encoded by the *PPIF* gene in humans showed that it is a highly basic protein. I was able to characterize it on a bi-dimensional gel of proteins from highly enriched mitochondrial fraction of cells. The intensity of the protein spot was quite considerable, which implies that cyclophilin (CyPD) is an abundantly expressed protein in the mitochondria. Recently, inhibition of CyPD by cyclosporin A in organs scheduled for transplantation stimulated some hopes that CsA could be crucial in stabilization and quality of such organs due to anti-ischemia action of CyPD/CsA complex. Diverse aspects of CyPD were analyzed by Drs. G.A. Porter, Jr., and G. Beutner [8]. FKBP51 was cloned in 1995, but this large immunophilin that has two FK506-like binding domains (FKBDs), three tetratricopeptide repeats (TPRs), and a calmodulin-binding domain, may have several different targets in the cell and may also be controlled by specific small molecular mass compounds. Complexes of this immunophilin with different small molecular mass synthetic ligands were reviewed by Drs. A. Hähle, S. Merz, C. Meyners, and F. Hausch [9], while its interaction with various proteins was analyzed by Drs. N.R. Zgajnar, S.A. De Leo, C.M. Lotufo, A.G. Erlejman, G. Piwien Pilipuk, and M.D. Galigniana [10]. PPIases have a vast phylogenetic distribution and following are three reviews on various aspects of this superfamily of proteins in plants, parasites, and prokaryotes. The importance of some PPIases on plant growth and survival was reviewed by Drs. I. Barbosa dos Santos and S.W. Park [11]. PPIases aid the folding of many large proteins such as collagen in mammalian organisms or some proteins that are crucial for the high quality of plant’ products in the agriculture. Although knowledge on functional features of PPIases in parasites remains scarce, the subject grew to a reasonable level and was presented by Drs. A.E. Perrone, N. Milduberger, A.G. Fuchs, P.L. Bustos, and J. Bua [12]. Some aspects of unusual types of PPIases, which are fusion proteins comprising both an FKBD and a cyclophilin-like domain (CLD), were reviewed by Dr. S. Barik [13]. Such fusion proteins comprising both, CLD and FKBP domains are typical only to some organisms at a low level of development. Some new facts in the research of a small family of PPIases, namely Pin1 and Pin14 and their control of some neoplasms, were reviewed by Dr. S. Stifani [14]. Pin1 preferentially isomerizes phosphorylated X-Pro epitopes. It was hypothesized that Pin1 could be a crucial factor in proper folding of some neuronal proteins, that in some cases may impair perfect functioning of the brain. It ends with my modest contribution to the analyses of two large sets (more than 15,000) of sequences of the cyclophilins and FKBPs, respectively [15]. *Nota bene* how the subject has evolved since 1989 when only partial sequences of FKBP12, human cyclophilin-A, and several other PPIases from yeast had been known. In this short technical note, I analyzed a large set of sequences of the cyclophilins and FKBPs that allowed me to set up several hypotheses, which probably deserve some attention and verification by future research on PPIases. 
